# Challenges and measures to improve interviewers’ bias in large-scale demographic surveys in India: Some suggestions based on analysis of NFHS-4 data

**DOI:** 10.1016/j.ssmph.2022.101104

**Published:** 2022-04-24

**Authors:** Tarun Kumar Roy, Rajib Acharya

**Affiliations:** a International Institute for Population Sciences, Mumbai, India; b Population Council, New Delhi, India

## Abstract

With increasing demand for more data at local level, the health surveys have expanded both their coverage and areas of inquiry. To cater to this demand, the sample size in National Family Health Surveys (NFHS) increased significantly and thereby raised concerns regarding quality. The present paper attempts to investigate the presence of interviewers' bias in the birth history data in 4^th^ round of NFHS in four states –Haryana, Odisha, Tamil Nadu and Maharashtra. The paper suggests a practical procedure that can be used to promote judicious supervision to minimize the non-sampling errors in future rounds of NFHS or other large-scale demographic surveys. Findings show that the outlier-based approach adopted in the paper helps in detecting the presence of interviewers’ bias in the enumeration of total children ever born as well as those born during 5 years prior to the survey – two critical variables in demographic surveys. Among the four study states, the extent of the bias was highest in Tamil Nadu. In fact, in Haryana, the data was found to be free of any bias in the recording of the occurrence of births in 5 years preceding the survey. It is suggested that it should be feasible to employ the outlier-based approach early when fieldwork is in progress, along with usual practice of generating field check tables. This approach would have the potential to not only streamline the supervision but also help salvage the data from any biasing effects. The biasing effects, if any and found early during fieldwork can be rectified by suitably arranging the necessary revisits to the respondents.

## 1 Introduction

Large-scale demographic surveys have increasingly gained popularity as an excellent source to obtain a variety of useful information from the population. They have been extensively used in developing countries. Initially, nationwide surveys focused mainly on understanding the levels of fertility and the factors associated with fertility. These surveys, known as the World Fertility Surveys, were undertaken in the 1970s. Later on, the scope of the surveys was broadened to include health-related issues, particularly maternal and child health-related ones. These surveys, which have been continuing since the 1980s, are popularly known as the Demographic and Health Surveys. India undertook its first such survey at the national level when it started the National Family Health Survey (NFHS-1) in 1992-93. The first three rounds of the survey, conducted approximately six years apart, estimated the parameters at the state-level. The state level estimates were based on approximately 4000 households, but with the number of states in the country varying between 20 and 30, the total sample size covered in these surveys hovered around 100,000 households. However, by maintaining a uniform strategy, the survey design was implemented independently at the state level. To complete the fieldwork within the stipulated time of about 3 months in a state, the survey design needed to engage about 30 interviewers; there was no major concern about the quality of the data.

In the 4^th^ round of the survey (NFHS-4, 2015-16), there was a major change in its design. Due to popular demand, the survey was undertaken again to provide estimates at a micro-level (district level). With more than 600 districts in the country, where most of the major states (with a population of at least 5 million) have at least 20 districts, the required sample size even at the state level often jumped to more than 20,000 households. For the country as a whole, the sample size for NFHS-4 has been more than 600,000 households (IIPS and ICF, 2017). To adhere to the requirement of completing the survey on time, the number of interviewers employed in a state often increased to around 100. This raised concern not only about the quality of selection of the interviewers but also regarding the necessary care required for their training and supervision of the fieldwork. Laxity in any of these components can increase the non-sampling errors and endanger the quality of data.

The non-sampling error depends largely on interviewers, how truthfully and effectively they are able to extract the required information from a respondent. This is true particularly when a large number of them are operating in the field. Apart from clarifying the doubts that an interviewer may have, in the initial stages, there is a need to have regular supervisory checks during fieldwork. But with a lengthy questionnaire and a large number of interviewers, the task becomes arduous and practically unmanageable. A feasible, yet effective way to expedite the work seemed to be to identify and focus on at least a few critical areas of inquiry that are not only important but are also prone to be mismanaged by the interviewers. Two such areas can be easily identified--one is the question of whether and how many births a woman has had in a given time (in NFHS-4, the time period was five years) preceding the survey. This information happens to be the basis for obtaining the recent level of fertility in a population. It is also linked with the estimation of the different parameters on receiving antenatal, natal, and postnatal care by a mother and also the child-care practices like feeding practices and immunization records. There are many questions that need to be filled for each child a woman has given birth to. If a respondent has not given any birth during five years preceding the survey, all these questions would be skipped. This could be a potent reason for misreporting the actual number of births a woman had within the interval, and it can introduce bias in the estimation of fertility as well as in the maternal and child health care indicators, which are of prime importance. The other is the question on the ever use of contraception discussed in an earlier paper (Roy, Porwal, & Acharya, 2021).

In addition to the selection of specific questions for monitoring, enforcing a periodic check (suggested in this paper) can simplify the supervision by focusing on a select group of interviewers who are likely to be engaged in such misuse. The procedure can go a long way in simplifying and improving the supervision and the data quality in a large-scale survey. It needs to be emphasized that the non-sampling errors are difficult to detect, and there is also no easy solution for it if detected after the completion of a survey. Since the suggested approach is implemented during fieldwork, it not only helps facilitate supervision but also enables taking remedial measures to remove the bias by revisiting some of the respondents.

The present paper tries to investigate the presence of interviewers’ bias in the birth history data in NFHS-4. It then suggests a procedure that can be used in the future to promote judicious supervision to minimize the non-sampling errors in large-scale demographic surveys.

## 2 Materials and methods

The birth history data in NFHS-4, which was conducted during 2015-16, provides quite a few important indicators. It gives the total number of children ever born to a woman, which is the basis for obtaining the cohort fertility. To avoid any confusion the total number of children by sex is enumerated separately for each woman as: a) total children staying with her, b) total children born but subsequently died, and c) children who are staying separately. A respondent, if simply asked her total children may not be aware of the purpose and might avoid mentioning her dead children or children who are staying separately. Interviewers are trained to be cautious about such possibilities and emphasize on the purpose clearly so as to avoid such misreporting. The birth history also provides for recording the number of children that were born during the five years preceding the survey. As mentioned before, this is a filter, wherein a large number of questions on maternal and child health care are skipped if a respondent is found to have no children in the preceding five years. Two types of errors in the data are visualized. One is the error present in the number of children ever born. Although, it is assumed that there is no recall bias as such in reporting the total number of births because a mother is not likely to forget about a child even if it was born long time ago. But still, she could be reluctant and think it is irrelevant, and hence she might not mention her dead children, if any or those staying separately and underreport her number of children ever born. There is a possibility of under enumeration in it if an interviewer is negligent and does not appropriately explain the purpose to the respondent. This is an error that occurs mainly due to the negligence of an interviewer, and it does not serve any purpose like saving interviewing time. The other is an error in reporting the number of children born during the previous five years. The inquiries on maternal and child health care are necessary for each of the birth that occurs in the given period. A woman can have either no birth or have a number of births during this period. Either by not reporting the occurrence of a birth altogether or by simply increasing the age of a child which is recorded in the birth history, an interviewer can underestimate the occurrence of a number of births within the interval to lessen her workload and speed up the interview. This is a type of error that is created deliberately. Both these errors are in fact biases because only under enumeration is envisioned. Since child adoption is negligible in the country, the overreporting of children is assumed to be nonexistent.

Outlier analysis helps us understand the existence of such biases among interviewers and segregates those who are likely to be involved in it. Regarding the negligence error, the analysis is essentially a comparison of mean children ever born between the interviewers, to understand who is likely to be underreporting it. In other words, to determine the interviewers whose recorded mean number of children ever born happens to be unduly below the overall mean children ever born recorded by all the interviewers to be regarded as outliers (McClave, Benson, & Terry, 2005). But the distribution of children ever born cannot be directly compared between two interviewers. For example, if a particular interviewer interviews a greater proportion of educated women and since educated women are likely to have fewer children, her mean children ever born is likely to be lower than another who has interviewed more uneducated women. There are quite a few other socio-economic and demographic characteristics that can influence the number of children ever born. There is a need to have a scale to standardize it by considering such characteristics that can influence the comparison. Multiple regression analysis helps get such a scale. The difference between the predicted value of children ever born from a regression and its observed value will provide the error that could be compared over the interviewers. Variables used in the regression are shown in Box 1.

Box 1

**Table:** 

Dependent variable (s)	Independent variables	Continuous/Dummies
Children ever born, total number of births in the last 5 years before the survey	Age at marriage	Continuous
	Level of education	0 – primary not completed, 1 – primary completed and above
	Religion	0- Hindu, 1- Others
	Caste	1 – SC, 0 – Others 1 – ST, 0 – Others 1 – General, 0 – Others Reference- OBC
	Whether has a son	1 – Yes,–0 - No
	Age at sterilization	1 – less than 30 years, 0 – Otherwise 1 – 30 years or above, 0 – Otherwise Reference – Nonuser of sterilization
	Parity at which used modern spacing method for the first time	1 – First at parity 0 or 1, 0 – otherwise 1 – First at parity 2 or above, 0 – Otherwise Reference – non-user of modern spacing method
	Two eldest children are girls	1 – Yes,–0 - No
	Wealth index category	1 – belongs to poorest or poor category, 0-otherwise 1 – belongs to rich or richest category, 0-otherwise Referen–e - Middle
	Marital duration	Continuous
	Square of marital duration	Continuous
	Child loss	1- Lost child, 0- otherwise
	Residence	1- Urban, 0- Rural

Alt-text: Box 1

For the analysis of identifying outliers, if any, among the interviewers, the regression approach helps obtain the error (e_ij_) committed by the i^th^ interviewer in the enumeration of children ever born for the j^th^ woman,

where, e_ij_ = estimated ceb (estimated value from the regression for the j^th^ woman) - ceb (as enumerated for j^th^ woman by the i^th^ interviewer).

It can be noted that if the i^th^ interviewer is negligent and does not properly emphasize the purpose of collecting the information on children ever-born her mean value of e_ij_ (for all j) denoted by m_i_ will tend to be positive and high. Let the limit beyond which she will be regarded as an outlier (and hence negligent) be L. Further, let M denote the overall mean of m_i_ and SD is the standard deviation of the means for all the interviewers (k).

L = M + 1.65 * SD/Sqrt(k)

A similar analysis is also used for detecting outliers in enumerating total number of births occurring for the 5 years preceding the survey.

Four different states have been considered for the illustration—Haryana, Odisha, Tamil Nadu and Maharashtra – from four different geographical regions of India. The analysis has been confined to women who are currently married and married only once. Since the analysis is essentially a comparison between interviewers, sample weights have not been used in the entire analysis.

### 2.1 Assessment of data quality in NFHS-4

Outliers in children ever born and number of children born in 5 years prior to the survey.

The regression analyses obtained for assisting detection of the outliers have been shown in Table 1a, Table 1ba and 1b for the two types of estimates. As mentioned, the regression basically serves the purpose of providing a standard scale to compare the performance of the interviewers. As such, the attention was on improving the goodness of fit of the regression. A few observations from the regression are worth mentioning. First, the goodness-of fit as shown by the value of the adjusted R^2^ is quite high considering the large number of observations available for model fitting. This is particularly true for the analysis of children ever born. The presence of son preference makes a significant influence on increasing the number of children born in all four states. Family size is increased in order to ensure that a son is born. Women having two consecutive daughters at the beginning of their reproductive life generally ends up having a large family size. The family size of a woman naturally depends on whether she has been using contraception. For example, the spacing method users who initiated the use early in their reproductive life, before having two children tended to have smaller family size in comparison to those who started using it after having two children.

**Table 1a:** Results of multiple regression analysis showing effects of socio-economic and demographic characteristics on children ever born in four states, NFHS-4.

Coefficients	Haryana	Odisha	Tamil Nadu	Maharashtra
Constant	0.760**	0.492**	0.743**	0.890**
Age at marriage	−0.014**	−0.017**	−0.018**	−0.028**
Primary or higher education	−0.266**	−0.184**	−0.083**	−0.217**
Non-Hindu	0.523**	0.337**	0.114**	0.329**
Scheduled caste	0.090**	0.145**	0.117**	−0.049**
Scheduled tribe	0.318**	0.167**	0.004^ns^	0.145**
General caste	−0.084**	−0.012^ns^	0.002^ns^	0.033**
Has a son	1.045**	1.035**	1.099**	1.045**
Had sterilization before 30 yrs age	−0.053**	0.040*	0.238**	0.260**
Had sterilization 30 yrs age or after	0.241**	0.536**	0.424**	0.578**
First used modern contraceptive at parity 0 or 1	−0.149**	−0.013 ^ns^	−0.012^ns^	0.070**
First used modern contraceptive at parity 2 or above	0.162**	0.259**	0.281**	0.342**
Two eldest children are girls	1.200**	1.171**	1.215**	1.219**
Belongs to poorest or poor category	0.297**	0.110**	0.078**	0.081**
Belongs to rich or richest category	−0.226**	−0.070**	−0.059**	−0.106**
Marital duration	0.095**	0.073**	0.042**	0.060**
Square of marital duration	−0.001**	−0.001**	−0.001**	−0.001**
Lost a child	1.219**	1.271**	1.084**	1.150**
Urban residence	−0.003^ns^	0.007 ^ns^	−0.033**	0.044**
Adj. R^2^	63.4	65.8	61.1	66.1
No. of women	16104	23686	20850	21537

Note: ns-not significant, *p < .05, **p < .01.

**Table 1b:** Results of multiple regression analysis showing effects of socio-economic and demographic characteristics on number of children born in five years preceding the survey among women who were not sterilized prior to 2011, in four states, NFHS-4.

Coefficients	Haryana	Odisha	Tamil Nadu	Maharashtra
Constant	1.187**	1.114**	1.067**	0.998**
Age at marriage	0.065**	0.051**	0.050**	0.060**
Primary or higher education	−0.089**	−0.043**	0.025^ns^	−0.001 ^ns^
Has a son	0.319**	0.200**	0.176**	0.241**
Two eldest children are girls	0.318**	0.243**	0.138**	0.208**
Belongs to poorest or poor category	0.010^ns^	0.024*	0.008^ns^	0.049**
Belongs to rich or richest category	−0.045*	0.008^ns^	0.001^ns^	−0.034*
Lost a child	0.060*	0.014 ^ns^	0.076**	0.010 ^ns^
Children ever born	0.270**	0.198**	0.240**	0.306**
Current age	−0.082**	−0.066**	−0.061**	−0.072**
Desire for no more children	−0.078**	−0.055**	−0.184**	−0.127**
Non-Hindu	−0.029^ns^	0.018 ^ns^	−0.034*	0.008 ^ns^
Urban	−0.024^ns^	−0.018 ^ns^	−0.011^ns^	−0.024*
Adj. R^2^	44.5	39.6	46.8	45.7
No. of women	10781	18803	12873	12424

Note: ns-not significant, *p < .05, **p < .01.

Results of the outlier analyses for children ever born are indicated in Table 2a. The presence of outliers is noticed in reporting children ever born in all four states. Its extent is higher in Tamil Nadu compared to other states.

**Table 2a:** Number of interviewers (who completed at least 20 interviews), number of interviewers deemed as outliers in recoding children ever born, number of once-married women interviewed by all the interviewers, and number of once-married women interviewed by outliers in four states, NFHS-4.

State	Number of interviewers	Number of interviewers deemed as Outliers	Total once-married women interviewed by all interviewers	Total once-married women interviewed by Outliers
Haryana	72	4 (5.5)	16104	906(5.6)
Odisha	119	7 (5.9)	23686	1070 (4.5)
Tamil Nadu	130	12 (9.2)	20850	2016 (9.7)
Maharashtra	137	16 (11.7)	21537	2037 (9.5)

The same is also true for number of children born 5 years before the survey (Table 2b). For this analysis, women who were reported as having accepted sterilization prior to 2011 were not included, as they did not have a birth during the 5 years period prior to the survey. Significantly, in Haryana, no outlier could be detected in its reporting. To what extent the deployment of a smaller number of interviewers in the state contributed to it is difficult to ascertain, but it seems, compared to Tamil Nadu, Maharashtra and Odisha, the data quality on children born in 5 years period prior to the survey has been better enumerated in this state. Although, both the parameters relate to the enumeration of children ever born, there is no overlap among the outliers. There is only one interviewer, in Tamil Nadu, who is found to be common as outlier in both the enumerations. In other words, among the interviewers who erred in the enumeration of total children ever born, practically none committed a similar mistake while enumerating children born in the last 5 years.

**Table 2b:** Number of interviewers (who completed at least 20 interviews) who interviewed women not sterilized prior to 2011, number of interviewers deemed as outliers in recoding number of children born in 5 years preceding survey, number of once-married women interviewed by all the interviewers, and number of once-married women interviewed by outliers in four states, NFHS-4.

State	Number of interviewers	Number of interviewers deemed as Outliers	Total once-married women interviewed by all interviewers	Total once-married women interviewed by Outliers
Haryana	72	0	10781	0 (0.0)
Odisha	117	4	18803	573 (3.0)
Tamil Nadu	130	19	12873	1970 (15.3)
Maharashtra	136	16	12424	1376 (11.1)

#### 2.1.1 Credibility of outlier analysis

The outlier analysis is supposed to help in isolating interviewers who are likely to be engaged in creating bias in the two parameters. To have a greater reliance on its ability to perform the task, few related parameters could be compared between the two groups: i) interviewers considered as outliers, and ii) interviewers whose reporting were found to be in line with what is expected, termed as ‘Normal’. Regarding children ever born, the following three indicators are compared between the two groups: percentage of reported dead children, percentage of reported children staying separately, and mean number of children ever born and surviving. The distribution of number of children born in 5 years preceding the survey has been considered for the other indicator. Table 3 clearly shows that the reporting of either the dead children or children living separately is lower among the outliers compared to the Normal interviewers. This is true in all four states. A chi-square test of significance reveals that the differences are statistically significant. The outliers were negligent in emphasizing the importance of these parameters to their respondents and underestimated the prevalence. A similar interpretation also emerges from Table 4a and Table 4b.

**Table 3:** Percent reporting dead and children staying separately among women who have at least one child by type of interviewer in four states, NFHS-4.

States	Percent reporting dead children			Percent reporting children staying separately		
	Outliers	Normal	Total	Outliers	Normal	Total
Haryana	6.7(821)	9.3(13759)	9.2 (14580)	8.2 (821)	13.0 (13759)	12.7 (14580)
Odisha	12.7(958)	16.7(20370)	16.5(21328)	16.5(958)	21.6(20370)	21.4(21328)
Tamil Nadu	3.9(1794)	6.7(17204)	6.5(18998)	13.2(1794)	17.8(17204)	17.4(18998)
Maharashtra	4.3(1842)	8.1(17551)	7.8(19393)	13.5(1842)	20.9(17551)	20.2(19393)

**Table 4a:** Mean Children ever born and surviving by type of interviewers in the two states, NFHS-4.

Age group	Haryana				Odisha			
	Outliers		Normal		Outliers		Normal	
	Ever born	Surviving	Ever born	Surviving	Ever Born	Surviving	Ever Born	Surviving
15–19	0.35	0.35 (20)	0.40	0.38 (331)	0.46	0.46(28)	0.41	0.39(699)
20–24	0.99	0.95 (129)	1.07	1.03 (2710)	1.02	0.99(173)	1.06	1.00 (3329)
25–29	1.67	1.64 (179)	1.88	1.80 (3385)	1.67	1.60(218)	1.74	1.64(4396)
30–34	2.17	2.12 (170)	2.45	2.35 (2703)	2.07	1.97(182)	2.33	2.15(4039)
35–39	2.44	2.37 (150)	2.80	2.67 (2282)	2.46	2.29(169)	2.72	2.48 (3705)
40–44	2.58	2.48 (132)	3.06	2.88 (2021)	2.55	2.38(159)	3.06	2.74(3229)
45–49	2.50	2.36 (126)	3.16	2.97 (1766)	2.64	2.36(141)	3.37	2.96(3123)
Total	2.01	1.95 (906)	2.25	2.14(21413)	1.98	1.86(1070)	2.28	2.08(22520)

**Table 4b:** Mean Children ever born and surviving by type of interviewers in the two states, NFHS-4.

Age group	Tamil Nadu				Maharashtra			
	Outliers		Normal		Outliers		Normal	
	Ever born	Surviving	Ever born	Surviving	Ever Born	Surviving	Ever Born	Surviving
15–19	0.37	0.37 (24)	0.44	0.42 (325)	0.46	0.45(65)	0.39	0.37(769)
20–24	1.05	1.04 (220)	1.12	1.09 (2280)	1.16	1.13(299)	1.21	1.17(3060)
25–29	1.53	1.50 (391)	1.66	1.61 (3730)	1.84	1.81(434)	1.90	1.84(3899)
30–34	1.83	1.79 (362)	2.00	1.94 (3426)	2.23	2.19(368)	2.38	2.30(3360)
35–39	1.98	1.93 (379)	2.23	2.14 (3434)	2.37	2.33(321)	2.61	2.52(3283)
40–44	1.89	1.84 (300)	2.29	2.19 (2782)	2.43	2.37(311)	2.88	2.74(2853)
45–49	1.87	1.83 (340)	2.39	2.25 (2857)	2.47	2.38(239)	3.05	2.89(2263)
Total	1.71	1.67 (2016)	1.94	1.87(18834)	2.01	1.97(2037)	2.21	2.12(19487)

With the decline in fertility over the years, it is expected that the mean children ever born would increase by age of women. In fact, a similar pattern can be seen among the women interviewed by the Normal group of interviewers in all four states and among outlier interviewers in Haryana, Odisha and Maharshtra. In Tamil Nadu, the mean number of children ever born increases continuously and reaches a level of 2.4 children among women aged 45–49 years. However, such a trend is not visible for women interviewed by the outliers. It shows an increasing trend for women up to the age of 35–39 years and then the mean number of children declines for the older women. It is interesting to note the age at which the reversal of the pattern takes place. A major cause for under-enumeration of children among the outliers is the underreporting of children who are staying separately. It is natural to expect that daughters of a woman or even sons who got married would be more likely to stay in a separate household; and only older women who have crossed the age 40 are likely to have such children who are themselves married. In other words, the dip in fertility after age 39 among the outliers is largely an outcome of the under enumeration of children staying separately by the outliers. This reinforces our faith in the procedure used for their selection. Again, the difference between the mean number of children ever born and surviving provides an idea about the number of dead children. For example, 3 percent of the children born to women aged 20–24 years interviewed by the ‘Normal’ interviewers died within approximately 9 years before the survey in Tamil Nadu. In fact, data on children ever born and surviving can be fruitfully used for the estimation of mortality levels, including infant mortality for smaller areas like at the district level (Mari Bhat, 1994). It can be seen from Table 4a, Table 4ba and 4b that the estimated proportion of children dying is lower among the outliers compared to the ‘Normal’ interviewers for all the age groups of women in all four states. This points towards an under-enumeration of dead children among the outliers as corroborated in Table 3.

The distribution of number of births reported by interviewers is shown in Table 5. In Haryana, no interviewer could be detected as outlier (Table 2b). In Odisha, Maharashtra and Tamil Nadu, where 4, 16 and 19 interviewers, respectively, were found to be outliers, a distinct difference in reporting of births could be seen between the ‘Outliers’ and ‘Normal’ interviewers. The outliers reported a much higher proportion of women without any birth during the interval (found to be statistically significant). Even with the distribution of a number of births among those who were reported having a birth, the proportion having two or more births was significantly lower among the outliers. In other words, there is a strong indication that the outliers, in all likelihood, indulged in underreporting of the occurrence of births to save time.

**Table 5:** Percentage distribution of women not sterilized prior to 2011 by number of children born in 5 years preceding survey by type of interviewers, NFHS-4.

States	Distribution of number of children born in preceding 5 years among interviewers deemed as Outliers				Distribution of number of children born in preceding 5 years among interviewers deemed as Normal			
	0	1	2+	Total	0	1	2+	Total
Haryana	--	--	--	--	48.6	34.2	17.2	100 (10781)
Odisha	59.0	34.0	7.0	100(573)	53.0	36.8	10.2	100(18121
Tamil Nadu	63.2	28.5	8.3	100.0(1970)	53.0	33.8	13.2	100 (10903)
Maharashtra	50.4	37.4	12.2	100(1376)	44.4	38.8	16.8	100(11025)

### 2.2 Improving supervision alongside fieldwork

The above discussion reveals the presence of interviewer bias in the data. The outlier analysis can be an excellent way to locate interviewers who are likely to be engaged in the biasing activities. To make any correction to the bias is however, difficult. Only possibility seems to be to trim the data, that is to exclude the data collected by the defaulting interviewers. But loss of data due to trimming can reduce the sample size available for valid estimation of some of the important parameters (eg., percent of women receiving antenatal, natal or postnatal care) for some affected districts. The procedure can help salvage the situation if it can detect such interviewers early during the fieldwork so that necessary action can be taken, and revisits to some of the women interviewed by them can be organized, if necessary. The moot point is, can this process of the outlier detection be made early during the fieldwork, and if yes, when to initiate it and at what interval should it be repeated? Two points need to be considered for this. Since the method is essentially a comparison of the mean estimated values of a parameter among the interviewers, it is better that each of them has the experience of completing at least 30 interviews. Assuming an average of 75 interviewers are employed in a survey, there will be enough data to conduct the regression also. This will require approximately two weeks, assuming that each interviewer can complete 3 interviews per day-- and provide sufficient data to carry out the analysis. Since the procedure might need a revisit to some of the women, there is another issue of how far they would have to travel for the revisits. Within 15 days they are likely to be in the same districts or at the most, in an adjoining one.

NFHS-4 data file provides information on number of interviews performed in each month of fieldwork. An attempt has been made to apply the procedure of outlier detection by considering the interviews performed during the 1^st^ month, then by using the cumulative number of cases at the end of the 2^nd^, 3^rd^, and so on in different months of fieldwork. For illustration, all the interviewers who have done at least 20 interviews have been included in the analysis. Table 6 shows the analysis for the four states for the reported number of children born in 5 years prior to the survey. Following few parameters are shown for each month of the analysis, in the table - total number of women interviewed, number of eligible interviewers, that is those who have completed at least 20 interviews, number of interviewers detected as outlier, and number of the outliers that are common with those found in the final analysis (based on the entire sample).

**Table 6:** Results of month-wise outlier analysis for reporting of number of children born during 5years preceding the survey in the four states, NFHS-4.

States	Parameters	Month of interview				
		1st	2nd	3rd	4th	5th
Haryana	Total no. of women interviewed	1870	4277	6849	9419	10760
	No. of eligible interviewers	54	71	72	72	72
	No. of interviewers detected as outliers	7	5	3	2	0
	No. of outliers who are also detected as outliers in the final analysis	0	0	0	0	0
Odisha	Total no. of women interviewed	4482	8071	11362	15103	18726
	No. of eligible interviewers	109	116	117	117	117
	No. of interviewers detected as outliers	14	12	9	10	4
	No. of outliers who are also detected as outliers in the final analysis	1	3	4	4	4
Tamil Nadu	Total no. of women interviewed	3628	7059	10526	12375	---
	No. of eligible interviewers	109	129	130	130	---
	No. of interviewers detected as outliers	23	23	21	19	---
	No. of outliers who are also detected as outliers in the final analysis	9	13	15	19	---
Maharashtra	Total no. of women interviewed	3757	6930	9379	11235	12404
	No. of eligible interviewers	94	130	132	136	136
	No. of interviewers detected as outliers	16	19	18	19	16
	No. of outliers who are also detected as outliers in the final analysis	6	10	12	15	16

The analysis shows the number of outliers to be high during the 1^st^ month and it declines gradually over different months of the fieldwork. A similar pattern is observed in all four states. In Haryana, the fieldwork continued for 5 months (in the sixth month a negligible number of interviews were done which are not included as it makes practically no difference to the interpretations). Even though no outliers could be detected while using the full sample, 7 of the 54 eligible interviewers were detected as outliers at the end of the 1^st^ month and it declined gradually to zero outliers during the last month. There are two components of the errors that lead to the detection of an interviewer as an outlier. One is the component called ‘error’ which signifies the deviation between the actual number of children a woman has during the interval and her predicted value for it on the basis of the regression that can occur by chance. The other, known as ‘bias’, denotes the difference that occurs due to deliberate underreporting of the number of children by some interviewers. The error component depends on the number of observations and tends to decline as the sample size increases, whereas bias does not depend on the sample size.

During the 1^st^ month, there will be quite a few interviewers with fewer completed interviews. Encountering a few unusual cases by such interviewers can render the error large enough to identify them as the outliers. The bias remains unaffected by the number of observations and hence such cases tend to appear again as outliers. This point emerges clearly from Table 6. As the survey progresses, the proportion of the biasing outliers—those who were detected as the outliers in the final analysis considering the entire sample—among the outliers detected in a month also increases. For example, in Tamil Nadu, the number of biasing outliers is 9 of 23 in the 1^st^, 13 of 23 in the 2^nd^ and 15 of 21 in the 3^rd^ month. A similar pattern is also observed in Odisha and Maharashtra. The presence of bias is less in Haryana, but two of the interviewers appeared as outliers in two different months, though they were not detected as the biasing interviewers. In Tamil Nadu, where the fieldwork lasted for four months, as many as 8 interviewers appeared to have created the bias continuously in all the 4 months and five more appeared in 3 of the 4 months (figures not shown in table). This shows that effective supervision lacked here and there was the presence of deliberate misreporting of births. Clearly, for such interviewers, timely supervision, and revisits to some of the women interviewed by them would have mitigated the problem.

## 3 Discussion

The technique of outlier detection—finding interviewers who are seemingly reporting a smaller number of children, either the children ever born or born during the 5 years preceding the survey, among a comparable group of interviewers—can facilitate having an efficient and effective supervision. It can isolate a smaller group of interviewers who are most likely to have been engaged in committing the bias. For both the parameters, the extent of the bias was higher in Tamil Nadu and Maharashtra. In both these states, about 10 percent of the women were interviewed by the defaulting interviewers, who underestimated the number of children ever born. The effect of such underestimation may not be negligible in the estimation of the cohort fertility and the level of mortality even at the state level, and it will also fail to provide any meaningful idea about them for many districts, where the sample size varies around only 1000 women. The efficiency of the interviewers is also found to be wanting in the estimation of the number of children born in 5 years preceding the survey, in these two states. For more than 15 percent of the women in Tamil Nadu, its estimation was likely to have been under-enumerated. This can endanger not only the estimation of the current level of fertility of the state but also influence the other parameters of the MCH care services. It is true that the state is doing well in controlling fertility as well as providing the MCH care services, but the correct assessment of the district level variations in providing such services could be largely affected.

A timely action to strengthen the supervision can go a long way in improving the quality of data collection. It is true that to effectively supervise more than 100 interviewers in an ongoing data collection is not easy. The outlier detection, if employed early during the data collection can be of considerable help in identifying a smaller group of interviewers who are in actual need for the attention. As indicated, this additional supervision can be initiated approximately after 15 days of the field work when adequate number of observations are available, and then it can be implemented again after the lapse of every 15 days. The analysis shows a uniform pattern in the occurrence of bias among the interviewers. In the earlier stage, a larger proportion of the interviewers are evaluated as an outlier, mainly due to the inadequacy of completing enough number of interviews. For example, about one-fifth of the interviewers were found to be an outlier in Tamil Nadu, after the 1st month of interview. With more interviews, in the successive stages, only those who are involved in committing the bias tends to remain. It needs to be emphasized that the supervising activity should not be regarded as a faultfinding exercise. Its sole purpose is to facilitate augmenting the quality of the data. An interviewer, apart from their own lack of motivation, may get involved in the biasing activity due to a variety of other reasons. Inadequacy of training along with insufficient field practice, insufficient remuneration and other facilities, proper work environment etc. can inhibit their performance (Roy & Pandey, 2008).

The value of ‘z’ for determining the limit of the interval beyond which the value of the mean children ever born by an interviewer is considered too low to regard her as an outlier can be suitably modified. If the interval is narrowed by reducing the limit, it would ensure greater confidence in the data in the sense that there would be less chance of missing interviewers involved in the biasing activities, but it would increase the cost of the survey as a higher proportion of the interviewers would require the supervisory check.

The exact mechanism to decide early as to which interviewer is involved in creating a bias and for whom it is a mere error that would disappear as they carry on, is a ticklish issue. The number of interviews completed by an interviewer can be one of the criteria for the decision. It seems reasonable to avoid making such a decision after the 1st exercise. The outliers detected during this stage need to be given additional training and necessary caution. In no case, however, the interviewers should be given any impression to suggest that there is under enumeration in the births. This can easily lead to a bias in the other direction. In fact, the procedure employed takes into account many other factors to judge what should be the reported number of births for a respondent. For example, an interviewer adjudged as an outlier may actually have a larger proportion of the births occurring during the interval compared to a ‘Normal’ interviewer. This can happen, for example, if an interviewer, regarded as an outlier, has interviewed a larger proportion of the younger women who are desirous to have additional children, compared to a ‘Normal’ interviewer. The proportion of children born during the interval, if compared without adjusting through the regression, can be higher among the former than that in the latter, but their adjusted proportion would be unduly lower to render them as an outlier.

Any interviewer who is found to be an outlier frequently, detected at least in two different occasions, would require a different treatment. Revisits of some of her cases can themselves work as a disincentive to engage in creating a bias. A mix of appropriate incentives and disincentives for the interviewers would go a long way in minimizing the extent of the bias and improving the data quality. It would be good to plan the field work in a manner so as to minimize the travel for the revisits. It is possible that the procedure might detect an interviewer as an outlier for the first time quite late during a fieldwork. For example, 4 of the 19 interviewers, in Tamil Nadu found to be involved in creating bias, were detected only at the end of the fieldwork, that is at the end of the 4th month. By that time, they had already completed sufficiently large number of interviews. For such cases, it would be desirable to revisit some of the interviews done by them recently, during the last month.

## 4 Conclusions

In conclusion, the paper shows that the outlier-based approach helps in detecting the presence of interviewers’ bias in the enumeration of total children ever born as well as those born during 5 years prior to the survey. The extent of the bias was higher in Tamil Nadu and Maharashtra than in Haryana or Odisha. In fact, in Haryana, the data was found to be free of any bias in the recording of the occurrence of births in 5 years preceding the survey. It is suggested that it should be feasible to employ the outlier-based approach early when fieldwork is in progress, along with usual practice of generating field check tables. This approach would have the potential to not only streamline the supervision but also help salvage the data from such biasing effects. The biasing effects, if any and found early during fieldwork can be rectified by suitably arranging the necessary revisits to the respondents.

## Author contribution

**TK Roy**: conceptualization and design of study, acquisition of data, analysis and interpretation of data, drafting the manuscript, revising the manuscript critically for important intellectual content, approved version of manuscript for publication; **R Acharya**: acquisition of data, drafting the manuscript, revising the manuscript critically for important intellectual content, approved version of manuscript for publication.

## Ethical statement

The authors used publicly available deidentified data for this analysis.
